# Rapid Identification of Black Grain Eumycetoma Causative Agents Using Rolling Circle Amplification

**DOI:** 10.1371/journal.pntd.0003368

**Published:** 2014-12-04

**Authors:** Sarah A. Ahmed, Bert H. G. Gerrits van den Ende, Ahmed H. Fahal, Wendy W. J. van de Sande, G. S. de Hoog

**Affiliations:** 1 Faculty of Medical Laboratory Sciences, University of Khartoum, Khartoum, Sudan; 2 CBS-KNAW Fungal Biodiversity Centre, Utrecht, The Netherlands; 3 Institute for Biodiversity and Ecosystem Dynamics, University of Amsterdam, Amsterdam, The Netherlands; 4 Mycetoma Research Centre, University of Khartoum, Khartoum, Sudan; 5 Department of Medical Microbiology and Infectious Diseases, Erasmus MC, Rotterdam, The Netherlands; 6 Peking University Health Science Center, Research Center for Medical Mycology, Beijing, China; 7 Sun Yat-Sen Memorial Hospital, Sun Yat-Sen University, Guangzhou, China; 8 Shanghai Institute of Medical Mycology, Changzheng Hospital, Second Military Medical University, Shanghai, China; 9 Basic Pathology Department, Federal University of Paraná State, Curitiba, Brazil; 10 King Abdulaziz University, Jeddah, Saudi Arabia; University of California San Diego School of Medicine, United States of America

## Abstract

Accurate identification of mycetoma causative agent is a priority for treatment. However, current identification tools are far from being satisfactory for both reliable diagnosis and epidemiological investigations. A rapid, simple, and highly efficient molecular based method for identification of agents of black grain eumycetoma is introduced, aiming to improve diagnostic in endemic areas. Rolling Circle Amplification (RCA) uses species-specific padlock probes and isothermal DNA amplification. The tests were based on ITS sequences and developed for *Falciformispora senegalensis, F. tompkinsii, Madurella fahalii, M. mycetomatis, M. pseudomycetomatis, M. tropicana, Medicopsis romeroi*, and *Trematosphaeria grisea*. With the isothermal RCA assay, 62 isolates were successfully identified with 100% specificity and no cross reactivity or false results. The main advantage of this technique is the low-cost, high specificity, and simplicity. In addition, it is highly reproducible and can be performed within a single day.

## Author Summary

Treatment of eumycetoma largely depends on the causative pathogen. Identification of mycetoma agent with phenotypic features is too limited, and physiological and biochemical techniques are laborious, time-consuming and nonspecific, whereas the currently available molecular methods based on DNA sequencing are specific but extremely expensive. We describe rolling circle amplification method for identification of black grain eumycetoma using species-specific padlock probes. Eight probes were designed and successfully used for species identification and the results were easily visualized in 1% agarose gel. RCA provides a simple, reproducible, and cost-effective method for rapid identification of mycetoma agent that can be used in low-resource clinical settings.

## Introduction

Black grain eumycetoma represents the most common fungal mycetoma worldwide. This chronic, erosive infection of subcutaneous tissues particularly affects the lower extremities and leads to severe disability [1]. The disease is considered a major health problem in tropical areas and is prevalent among people of low socio-economic status [2].

Mycetoma presents as a subcutaneous mass with multiple sinuses that discharge pus, serous fluid and grains, i.e. the characteristic compact grains of the causative agent formed inside the lesion [3].

A wide range of microorganisms has been reported to cause mycetoma. For treatment, not only differentiation between (fungal) eumycetoma and (bacterial) actinomycetoma is important, but also the identity of the causative agent, since species differ in their response to antimicrobial drugs [4]. In endemic countries, clinical diagnosis may be the only diagnostic method. A fully developed mycetoma lesion is easily identified clinically, whereas in early stages with the absence of grains, the infection may be confused with phaeomycosis or soft tissue tumors [1]. In such cases fine needle aspiration cytology or deep surgical biopsy for histological examination are useful [1], [5]. Some fungal and bacterial grains have a characteristic histological appearance which helps in provisional identification, but recognition of the causative species remains impossible [6]. Isolation of the pathogen from discharged grains or from biopsies allows identification of agents that sporulate, but most of the species lack phenotypic characteristics [3]. Molecular techniques have been introduced to facilitate the identification of nondescript organisms [7], [8], [9], but are of high cost and time-consuming. Thus, there is a need for a fast, simple and reliable method for identification.

Rolling circle amplification (RCA) is a powerful diagnostic method based on detection of specific nucleic-acid sequences and enzymatic amplification of circularized oligonucleotide probes under isothermal conditions [10]. The probes are linear oligonucleotides that contain two target-complementary sequences at their ends joined by linkers [11]. The ends of the probe hybridize to the complementary target in juxtaposition and then ligate which allows the circularization of the probe [11]. The circular structured molecule then amplifies with DNA polymerase that has strand displacement and progressive DNA synthesis activity resulting in series of repeats of the original circular template [11], [12]. The technique has been proven to be rapid, specific and low-cost for molecular identification of viruses, bacteria, and fungi [13], [14], [15], [16]. It has been applied for identification of a rare black grain mycetoma species *Exophiala jeanselmei* [17]. In addition, RCA has been used successfully for identification of white grain mycetoma species *Scedosporium boydii* [18]. The aim of the present study is to develop RCA-based diagnostics for the most common agents of black-grain eumycetoma.

## Materials and Methods

### Strains analyzed

The study included 62 isolates belonging to eight species causing black grain mycetoma: *Madurella mycetomatis* (n = 32), *M. fahalii* (n = 1), *M. pseudomycetomatis* (n = 3), *M. tropicana* (n = 2), *Trematosphaeria grisea* (n = 10), *Falciformispora senegalensis* (n = 6), *F. tompkinsii* (n = 2), and *Medicopsis romeroi* (n = 6). Strains were obtained from the reference collections of CBS-KNAW Fungal Biodiversity Centre (Utrecht, The Netherlands) and the Mycetoma Research Centre (MRC, Khartoum, Sudan) and are listed with metadata in Table 1. Type strains of all tested species were included. All strains were identified down to species level by sequencing of the rDNA ITS region [19], [20].

**Table 1:** Isolation source, origin, of strains analyzed.

	Name	No.	Source	Origin
1.	*Falciformispora senegalensis*	CBS 196.79 ^T^	Mycetoma	Senegal
2.	*Falciformispora senegalensis*	CBS 197.79	Human	Senegal
3.	*Falciformispora senegalensis*	CBS 198.79	Mycetoma	Senegal
4.	*Falciformispora senegalensis*	CBS 199.79	Human	Senegal
5.	*Falciformispora senegalensis*	CBS 132257	Mycetoma	Sudan
6.	*Falciformispora senegalensis*	CBS 132272	Mycetoma	Sudan
7.	*Falciformispora tompkinsii*	CBS 200.79	Man	Senegal
8.	*Falciformispora tompkinsii*	CBS 201.79	Man	Senegal
9.	*Medicopsis romeroi*	CBS 252.60 ^T^	Mycetoma	Venezuela
10.	*Medicopsis romeroi*	CBS 132878	Mycetoma	India
11.	*Medicopsis romeroi*	CBS 122784	Plant	
12.	*Medicopsis romeroi*	CBS 123975	Phaeohyphomycosis	India
13.	*Medicopsis romeroi*	CBS 128765	Subcutaneous cyst	Kuwait
14.	*Medicopsis romeroi*	CBS 135987	onychomycosis	Netherlands
15.	*Trematosphaeria grisea*	CWZ 29591		
16.	*Trematosphaeria grisea*	CBS 332.50^ T^	Mycetoma	Chili
17.	*Trematosphaeria grisea*	CBS 246.66	Submandibular abscess	India
18.	*Trematosphaeria grisea*	CBS 120271	Tap water	The Netherlands
19.	*Trematosphaeria grisea*	CBS 135982	Pastry gel	The Netherlands
20.	*Trematosphaeria grisea*	CBS 135984	Water	The Netherlands
21.	*Trematosphaeria grisea*	CBS 136543	Water	The Netherlands
22.	*Trematosphaeria grisea*	CBS 135985	Water	The Netherlands
23.	*Trematosphaeria grisea*	CBS 135983	Water	The Netherlands
24.	*Trematosphaeria grisea*	CBS 136537	Water	The Netherlands
25.	*Madurella mycetomatis*	CBS 132258 (Mm10)	Mycetoma	Sudan
26.	*Madurella mycetomatis*	CBS 132259 (Mm13)	Mycetoma	Sudan
27.	*Madurella mycetomatis*	CBS 132260 (Mm14)	Mycetoma	Sudan
28.	*Madurella mycetomatis*	CBS 132261 (Mm16)	Mycetoma	Sudan
29.	*Madurella mycetomatis*	CBS 132262 (Mm18)	Mycetoma	Sudan
30.	*Madurella mycetomatis*	CBS 132263 (Mm22)	Mycetoma	Sudan
31.	*Madurella mycetomatis*	CBS 132265 (Mm28)	Mycetoma	Sudan
32.	*Madurella mycetomatis*	CBS 132266 (Mm29)	Mycetoma	Sudan
33.	*Madurella mycetomatis*	CBS 132267 (Mm30)	Mycetoma	Sudan
34.	*Madurella mycetomatis*	CBS 132269 (Mm33)	Mycetoma	Sudan
35.	*Madurella mycetomatis*	CBS 132270 (Mm36)	Mycetoma	Sudan
36.	*Madurella mycetomatis*	CBS 132273 (Mm44)	Mycetoma	Sudan
37.	*Madurella mycetomatis*	CBS 132274 (Mm45)	Mycetoma	Sudan
38.	*Madurella mycetomatis*	CBS 132285 (Mm46)	Mycetoma	Sudan
39.	*Madurella mycetomatis*	CBS 132276 (Mm49)	Mycetoma	Sudan
40.	*Madurella mycetomatis*	CBS 109814 (Mm54)	Mycetoma	Sudan
41.	*Madurella mycetomatis*	CBS 131320 (Mm55)	Mycetoma	Sudan
42.	*Madurella mycetomatis*	CBS 132280 (Mm58)	Mycetoma	Sudan
43.	*Madurella mycetomatis*	CBS 132281 (Mm63)	Mycetoma	Sudan
44.	*Madurella mycetomatis*	CBS 132282 (Mm64)	Mycetoma	Sudan
45.	*Madurella mycetomatis*	CBS 132283 (Mm68)	Mycetoma	Sudan
46.	*Madurella mycetomatis*	CBS 132284 (Mm71)	Mycetoma	Sudan
47.	*Madurella mycetomatis*	CBS 132285 (Mm72)	Mycetoma	Sudan
48.	*Madurella mycetomatis*	CBS 132286 (Mm73)	Mycetoma	Sudan
49.	*Madurella mycetomatis*	CBS 132287 (Mm78)	Mycetoma	Sudan
50.	*Madurella mycetomatis*	CBS 132288 (Mm83)	Mycetoma	Sudan
51.	*Madurella mycetomatis*	CBS 109801^T^	Mycetoma	Sudan
52.	*Madurella mycetomatis*	CBS 110087	Mycetoma	Sudan
53.	*Madurella mycetomatis*	CBS 110359	Mycetoma	Mali
54.	*Madurella mycetomatis*	CBS 110356	Mycetoma	Mali
55.	*Madurella mycetomatis*	CBS 132419	Mycetoma	India
56.	*Madurella mycetomatis*	CBS 132589	Mycetoma	India
57.	Madurella *tropicana*	CBS 201.38 ^T^	Mycetoma	Indonesia
58.	Madurella *tropicana*	CBS 331.50		
59.	Madurella *pseudomycetomatis*	CBS 129177 ^T^	Mycetoma	China
60.	Madurella *pseudomycetomatis*	CBS 216.29	Mycetoma	
61.	Madurella *pseudomycetomatis*	CBS 248.48	Mycetoma	New Mexico
62.	Madurella *fahalii*	CBS129176 ^T^	Mycetoma	Sudan

(CBS Centraalbureau voor Schimmelcultures; Between brakets Erasmus collection number for strains from Sudan; Type strains marked with^T^)

### DNA extraction and target amplification

DNA was extracted using cetyltrimethyl ammonium bromide (CTAB) method as described by Möller et al. [21]. Amplification of the ITS region was performed using primers V9G and LS266 [22] in a 25 µL reaction mixture containing: 10 ng of template DNA, 0.1 mM each dNTP, 0.6 U Taq polymerase (GC Biotech, Alphen aan den Rijn, The Netherlands), 1 µL of each primer (10 pmol) and 2.5 µL reaction buffer (0.1 M Tris-HCl, 0.5 M KCl, 25 mM MgCl_2_, 0.1% gelatin, 1% Triton X-100). PCR reactions consisted of a 5 min predenaturation step at 95°C, followed by 30 cycles of 95°C for 30 s, 52°C for 30 s and 72°C for 1 min, with final post elongation step at 72°C for 7 min. PCR products were detected by electrophoresis using 1% agarose gels.

### Padlock probe design

Sequences of the ITS region were used to design 8 probes specific for each species used in this study. Two alignments were generated since the analyzed species were known to belong to two different fungal orders [19], [20]. ITS derived from *Madurella* (*Sordariales*) were aligned with 200 isolates of *Chaetomiaceae* including *Chaetomium*, *Thielavia*, and *Achaetomium*. For the remaining species (*Pleosporales*) an alignment was constructed to include representative isolates of the family *Trematosphaeriaceae* and of coelomycetes in the suborder *Pleosporineae*. Sequences were aligned using BioNumerics v4.61 (Applied Maths, Sint-Martens-Latem, Belgium). Probes were designed with minimum secondary structure and were checked using PrimerSelect (DNASTAR Lasergene, WI, U.S.A.). To insure specificity of the probes, target-specific sites of each padlock probe was submitted to BLAST in NCBI sequence database for homologous sequences. The melting temperature of the 5′ end of the probe binding arm was designed to be>63°C while for the 3′ end binding arm it was designed to be at least 15°C below the annealing temperature. The probes were phosphorylated at the 5′ end and are listed in Table 2. Probe linkers were taken from Zhou et al. [23].

**Table 2:** Oligonucleotide padlock probes and probe-specific primers used for species identification with RCA.

Species name	Probe and primer name	Sequences
*M. tropicana*	MTROP	5′pGAGAGCAAACAGGGTGTTGTATAgatcaTGCTTCTTCGGTGCCCATtacgaggtgcggatagctacCGCGCAGACACGATAgtctaCAGAGAGGCCATA-3′
*M. pseudomycetomatis*	MPSEU	5′pGGAGCAACAGGGTGTTGTATAATgatcaTGCTTCTTCGGTGCCCATtacgaggtgcggatagctacCGCGCAGACACGATAgtctaAGAGAGGCCATAC -3′
*M. fahalii*	MFAH	5′pTGATACTACTACGCTCGGAGTGACgatcaTGCTTCTTCGGTGCCCATtacgaggtgcggatagctacCGCGCAGACACGATAgtctaCCCTGAGCGAGG -3′
*T. grisea*	TGRIS	5′pACCCGTAGGTCCTCCCAAAAGCGgatcaTGCTTCTTCGGTGCCCATtacgaggtgcggatagctacCGCGCAGACACGATAgtctaTGGACGCCAGTCC-3′
*F. senegalensis*	FSEN	5′pACATAGACAAGGGTGTTGCCGGCgatcaTGCTTCTTCGGTGCCCATtacgaggtgcggatagctacCGCGCAGACACGATAgtctaCAACGTACGGTAC-3′
*F. tompkinsii*	FTOM	5′pTCTTCCCAAAGTGCGCAAAGTGCgatcaTGCTTCTTCGGTGCCCATtacgaggtgcggatagctacCGCGCAGACACGATAgtctaCTATGCCACCAAG-3′
*M. romeroi*	MRO	5′pAAGGCGAGTCCACGCACTCTGGgatcaTGCTTCTTCGGTGCCCATtacgaggtgcggatagctacCGCGCAGACACGATAgtctaCTGCCAATGACTTT -3′
*M. mycetomatis*	MYC	5′pACTACACTACCGGGAGGCCCgatcaTGCTTCTTCGGTGCCCATtacgaggtgcggatagctac CGCGCAGACACGATAgtctaAGGGGGCCGAGGGAC-3′
	RCA1	5′-ATGGGCACCGAAGAAGCA-3′
	RCA2	5′-CGCGCAGACACGATA-3′

5′p- indicate phosphorylation of 5′ end, probes binding arms are underlined, the arms joined with non specific region lower case and RCA1 and RCA2 primer binding regions are bolded.

### Probe ligation

Padlock probe ligation was performed in a mixture consisting of 1 µl purified ITS amplicons, 2 U pfu DNA ligase (Epicentre Biotechnologies, Madison, WI, U.S.A.), and 0.1 µmol/l padlock probe in buffer (20 mM Tris-HCl pH 7.5, 10 mM MgCl_2_, 20 mM KCl, 0.1% Igepal, 0.01 mM rATP, 1 mM DTT), with a total reaction volume of 10 µl. Ligation conditions were: 5 min denaturation at 94°C, followed by 7 cycles of 94°C for 30 sec, 63°C for 4 min, and final cooling at 10°C.

### Exonucleolysis

Prior to RCA amplification reaction and in order to reduce the ligation-independent amplification, ligation products were treated by addition of 10 U exonucleases I and 10 U exonucleases III (New England Biolabs, Hitchin, U.K.) with a final volume of 20 µl. The mixture was then incubated for 30 min at 37°C, followed by 3 min at 94°C to deactivate the endonuclease enzymes.

### Rolling circle amplification (RCA)

RCA amplification reaction was performed in a 50 µl mixture containing; 2 µl ligation product, 8 U *Bst* DNA polymerase (New England Biolabs), 10 pmol of each RCA primer (Table 2), and 400 µM dNTP mix. The mixture was incubated at 65°C for 60 min and cooled at 10°C. Electrophoresis on a 1% agarose gel was used to visualize RCA products. A positive reaction is indicated by the presence of ladder-like pattern. The reaction was also visualized by adding 1.0 µl of a 10-fold diluted SYBR Green I (Cambrex BioScience, Workingham, U.K.) to 10 µl of the amplification product. Accumulated double stranded DNA was detected with UV transilluminator (Vilber Lourmat, Marne-la-Vallée, France).

### Determination of analytical specificity and sensitivity

The specificity of the 8 RCA probes was tested using strains of black-grain mycetoma causative species listed in table 1. Analytical sensitivity was determined using 10-fold serial dilution of *M. mycetomatis* (CBS 109801) and *M. fahalii* (CBS 129176) DNA and the test was performed as mentioned above. In addition, RCA was performed directly using DNA samples without amplification of the target gene. To evaluate the detection limit from direct DNA samples two-fold serial dilutions of target DNA were tested.

The sensitivity of the RCA probes was also determined by 10-fold serial dilution of MYC and MFAH probes tested with amplified ITS of *M. mycetomatis* and *M. fahalii* respectively.

## Results

RCA was used to identify 62 strains belonging to eight species causing human eumycetoma. Since black grain eumycetoma species are known to be phylogenetically distant, it is easy to find unique sites for their identification. The ribosomal ITS region was sufficient for identification of all species and showed no intraspecific variability within a set of 100 *M. mycetomatis* strains in our collection. For *M. mycetomatis*, *M. tropicana*, *M. pseudomycetomatis*, and *F. senegalensis* the ITS1 region was selected for probe design, while for *M. fahalii, T. grisea*, *F. tompkinsii* and *M. romeroi* the ITS 2 region was found to be more suitable.

**Figure 1:**
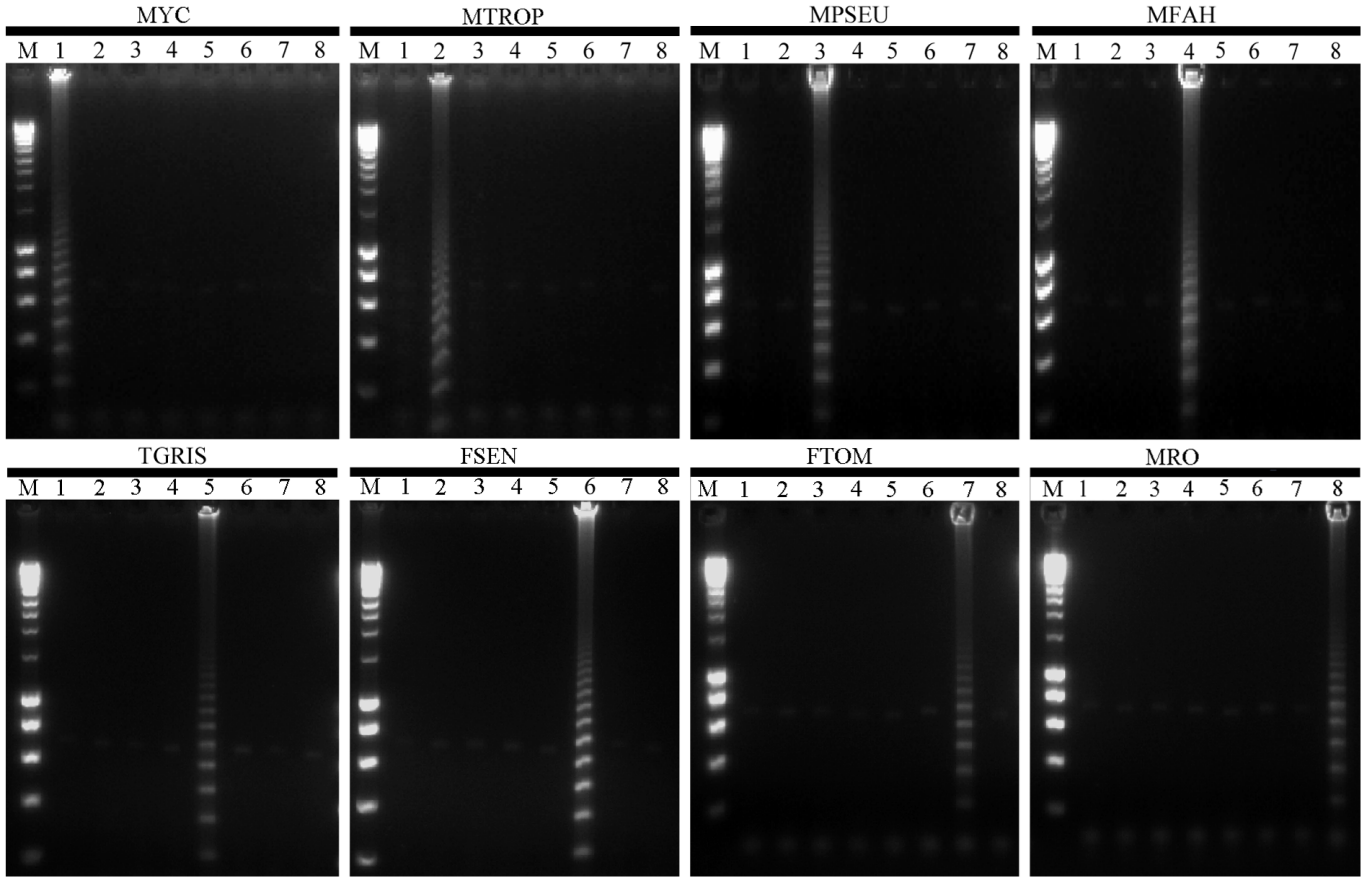
Specificity of rolling circle amplification probes. Agarose gel electrophoresis analysis of rolling circle amplification products. Positives probe signal seen as band pattern was only present with matched template–probe mixtures. Probe names are indicated on the top of the gel. Lanes; 1* M. mycetomatis* CBS 109801^T^, 2 *M. tropicana* CBS 201.38^T^, 3 *M. pseudomycetomatis* CBS 129177^T^, 4 *M. fahalii* CBS 129176^T^, 5 *T. grisea* CBS 332.50^T^, 6 *F. senegalensis* CBS 196.79^T^, 7 *F. tompkinsii* CBS 200.70, 8* M. romeroi* CBS 252.60^T^, M DNA ladder.

RCA results for the tested strains were easily visualized in 1% agarose gel. Positive reactions demonstrated ladder like patterns while negative reactions resulted in a clear background (Fig. 1). With SYBR green, positive results showed green fluorescence when exposed to UV light, while negatives did not. When exonucleolysis was performed some inhibition was observed with low RCA positive signals on gel or with fluorescence. Faint non-specific bands were observed when this step was omitted. RCA reactions were performed successfully without digestion with exonucleases, as the non-specific bands did not interfere with RCA results.

**Figure 2:**
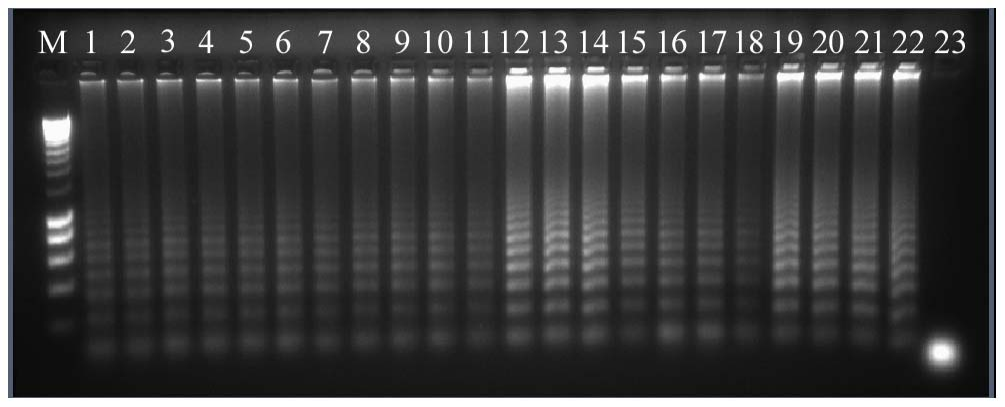
*Madurella mycetomatis* identification by RCA. Gel representation of rolling circle amplification reaction using *Madurella mycetomatis* probe (MYC) for strains recovered from mycetoma patient of origin: lane 1–18 Sudan; lane 19, 20 Mali; lane 21, 22 India; lane 23 negative control water; lane M ladder.

All *M. mycetomatis* strains were correctly identified with RCA, irrespective of their geographical origin (Sudan, India, Mali) (Fig. 2). For the other agents, each individual species-specific probes yielded positive results with their corresponding species and with 100% agreement with ITS sequencing (Fig. 2, Table 3). No cross reactivity or false positive and negative results were observed. The sensitivity of RCA when using amplified product of the target gene was less than 32×10^−3^ ng of DNA. A higher concentration of 100 ng is needed when the test is carried out directly from the DNA samples without amplification of the ITS. The probes were very sensitive and a concentration of 6.6×10^−5^ ng was successfully ligated and then amplified with RCA.

**Table 3:** Rolling circle amplification results of analysed strain.

Strains	RCA result for species-specific padlock probe							
	MYC	MTROP	MPSEU	MFAH	TGRIS	FSEN	FTOM	MRO
*M. mycetomatis* (32)	+	-	-	-	-	-	-	-
*M. tropicana* (2)	-	+	-	-	-	-	-	-
*M. pseudomycetomatis* (3)	-	-	+	-	-	-	-	-
*M. fahalii*(1)	-	-	-	+	-	-	-	-
*T. grisea* (10)	-	-	-	-	+	-	-	-
*F. senegalensis*(6)	-	-	-	-	-	+	-	-
*F. tompkinsii* (2)	-	-	-	-	-	-	+	-
*M. romeroi* (6)	-	-	-	-	-	-	-	+

Positive results (+), negative results (-).

**Figure 3:**
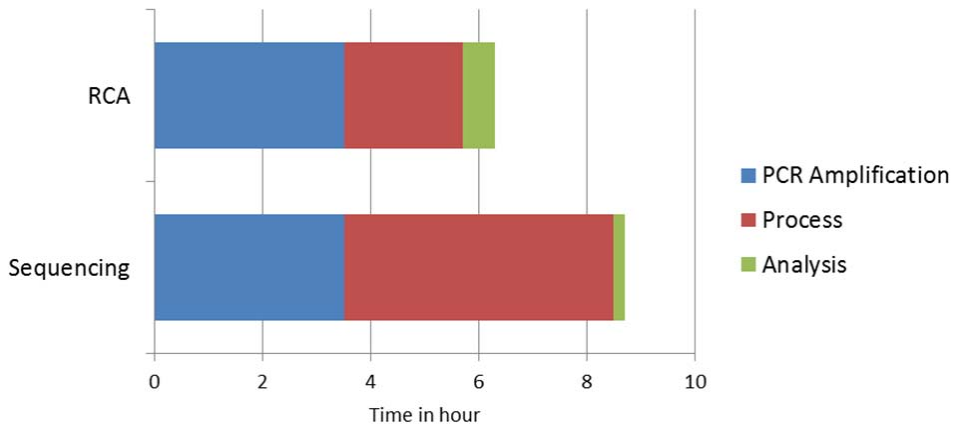
Identification time of species using rolling circle amplification (RCA) and sequencing of ITS.

The turnaround time required for conducting the entire experiment including PCR amplification of target DNA, RCA processing and analysis was found to be 6 hours. DNA sequencing of the ITS region took more than 8 hours to be performed (Fig. 3).

## Discussion

Mycetoma is a unique tropical disease, endemic in many tropical and subtropical regions that has been recently added to the WHO list of neglected tropical diseases. [24]. It is mainly prevalent in what is known as “mycetoma belt” which includes Mexico, Senegal, Sudan, India and other countries between tropic of cancer [1]. In 2014, a mycetoma consortium of scientists and physicians published research gaps on mycetoma which need to be addressed in the coming years [2]. One of the research priorities identified was the need to develop a reliable and cost-effective method for species identification to improve diagnosis [2].

Mycetoma agents have been extensively studied in recent years [8], [9], [20]. The large phylogenetic distance between a number of these agents provides the possibility to use a moderately variable marker like rDNA ITS for species identity. Ahmed et al. [25] developed PCR-restriction fragment length polymorphism (RFLP) for identification of *M. mycetomatis* targeting the ITS region. However, with the description of the molecular siblings *M. fahalii*, *M. pseudomycetomatis*, and *M. tropicana* [26] the method might be insufficiently accurate. Moreover, there is a need for identification these siblings species; *Madurella grisea* appeared to be distantly related and was re-named as *T. grisea* [20].

In the present study we developed a simple, fast and highly specific molecular method for the identification of agents of black grain mycetoma. In this method, the ITS region is easily amplified using one set of primers, which simplifies the use. In a second, isothermal amplification reaction padlock probes are used to identify the species by RCA. The only equipment necessary is a thermocycler for the PCR reaction and a water bath or heating block for the RCA reaction. This relative simplicity enhances possible use in routine laboratories in endemic areas. Due to its robustness, high potential, and reproducibility, RCA is increasingly used as a diagnostic tool in pathogenic fungi, e.g. agents of chromoblastomycosis, dermatophytes, *Aspergillus, Candida,* and *Talaromyces marneffei* [16], [23], [27], [28]. The method does not require DNA sequencing and is therefore considered as a rapid and cost-effective. Applications are being expanded to nano- and biotechnology [29].

In the present study eight species-specific probes were designed and used for identification of 62 isolates. For the RCA reaction species probe hybridization to the 3′ and 5′ ends of target DNA and joining of adjacent ends by DNA ligase when both show perfect complementarity. The ligation appears to be highly specific and thus the method can detect single nucleotide polymorphism [30]. The amplification reaction is driven by an isothermal DNA polymerase to amplify the circularized probes with high efficiency and an estimated capacity to synsthesize more than 70,000 bp per hour [31]. RCA products can be detected with different methods including gel electrophoresis, radiolabeling, UV absorbance, fluorescence, and single molecule detection [32]. It was known that the positive signals can be detected within 15 min after starting the RCA reaction by real time PCR [23]. In the present study the RCA positive signal was easily visualized using both gel electrophoresis and fluorescent dye. The duration of our RCA protocol was 2 h, but additional time is required for DNA extraction and ITS amplification. Compared to the DNA sequencing the turnaround time for RCA is 2 hours less than sequencing and this even more if there is no in-house sequencer available.

Our results with eight padlock probes showed that RCA accurately identified all species with no cross reactivity (Fig. 1). It may be concluded that RCA is extremely useful for specific identification of agents of mycetoma. Performance and rapid turnaround time features make the RCA suitable for quick and reliable diagnosis, which is an enormous improvement compared to the current phenotypic identification of mostly non-sporulating cultures. Future application of RCA could be the detection of agents DNA directly from clinical samples without requirement of culturing.

## Supporting Information

S1 Figure STARD flowchart for RCA.
(PDF)

## References

[1] Fahal AH (2006) Mycetoma, Clinicopathological Monograph. Khartoum: Khartoum University Press.

[2] van de Sande WW, Maghoub el S, Fahal AH, Goodfellow M, Welsh O, et al (2014) The mycetoma knowledge gap: identification of research priorities. PLoS Negl Trop Dis. 8: e2667. 24675533 10.1371/journal.pntd.0002667 PMC3967943

[3] Kwon-Chung KJ, Bennet JE (1992) Medical Mycology. Philadelphia: Lea and Febiger.

[4] Fahal AH (2010) Management of mycetoma. Expert Rev Dermatol 5: 87–93.

[5] Yousif BM, Fahal AH, Shakir MY (2010) A new technique for the diagnosis of mycetoma using fixed blocks of aspirated material. Trans R Soc Trop Med Hyg 104: 6–9. 19700179 10.1016/j.trstmh.2009.06.015

[6] Alam K, Maheshwari V, Bhargava S, Jain A, Fatima U, et al (2009) Histological diagnosis of madura foot (mycetoma): a must for definitive treatment. J Glob Infect Dis 1: 64–67. 20300390 10.4103/0974-777X.52985 PMC2840937

[7] de Hoog G S, Buiting A, Tan C S, Stroebel A B, Ketterings C, et al (1993) Diagnostic problem with imported cases of mycetoma in The Netherland. Mycoses 36: 81–87. 8366880 10.1111/j.1439-0507.1993.tb00693.x

[8] Desnos-Ollivier M, Bretagne S, Dromer F, Lortholary O, Dannaoui E (2006) Molecular identification of black-grain mycetoma agents. J Clin Microbiol 44: 3517–3523. 17021076 10.1128/JCM.00862-06 PMC1594755

[9] Ahmed AO, Desplaces N, Leonard P, Goldstein F, De Hoog S, Verbrugh H, et al (2003) Molecular detection and identification of agents of eumycetoma: detailed report of two cases. J Clin Microbiol. 41: 5813–5816. 10.1128/JCM.41.12.5813-5816.2003 PMC309011 14662990

[10] Gilbert W, Dressler D (1968) DNA Replication: The Rolling Circle Model. Cold Spring Harb Symp Quant Biol 33: 473–484. 4891987 10.1101/sqb.1968.033.01.055

[11] Nilsson M, Malmgren H, Samiotaki M, Kwiatkowski M, Chowdhary BP, et al (1994) Padlock probes: circularizing oligonucleotides for localized DNA detection. Science 265: 2085–2088. 7522346 10.1126/science.7522346

[12] Inoue J, Shigemori Y, Mikawa T (2006) Improvements of rolling circle amplification (RCA) efficiency and accuracy using *Thermus thermophilus* SSB mutant protein. Nucleic Acids Res 34: e69. 16707659 10.1093/nar/gkl350 PMC1463899

[13] Wang B, Potter SJ, Lin Y, Cunningham AL, Dwyer DE, et al (2005) Rapid and sensitive detection of severe acute respiratory syndrome Coronavirus by rolling circle amplification. J Clin Microbiol 43: 2339–2344. 15872263 10.1128/JCM.43.5.2339-2344.2005 PMC1153787

[14] Macera L, Cortey M, Maggi F, Segalés J, Kekarainen T (2011) A novel rolling circle amplification assay to detect members of the family *Anelloviridae* in pigs and humans.Virus Res. 160: 424–427. 10.1016/j.virusres.2011.06.025 21762734

[15] Chen X, Wang B, Yang W, Kong F, Li C, et al (2014) Rolling circle amplification for direct detection of rpoB gene mutations in *Mycobacterium tuberculosis* isolates from clinical specimens. J Clin Microbiol 52: 1540–1548. 24574296 10.1128/JCM.00065-14 PMC3993705

[16] Najafzadeh MJ, Sun J, Vicente VA, de Hoog GS (2011) Rapid identification of fungal pathogens by rolling circle amplification using Fonsecaea as a model. Mycoses 54: e577–e582. 21910759 10.1111/j.1439-0507.2010.01995.x

[17] Najafzadeh MJ, Dolatabadi S, Saradeghi Keisari M, Naseri A, Feng P, et al (2013) Detection and identification of opportunistic *Exophiala* species using the rolling circle amplification of ribosomal internal transcribed spacers. J Microbiol Methods 94: 338–342. 23872449 10.1016/j.mimet.2013.06.026

[18] Lackner M, Najafzadeh MJ, Sun J, Lu Q, Hoog GS (2012) Rapid identification of *Pseudallescheria* and *Scedosporium* strains by using rolling circle amplification. Appl Environ Microbiol 78: 126–133. 22057865 10.1128/AEM.05280-11 PMC3255646

[19] de Hoog GS, Ahmed SA, Najafzadeh MJ, Sutton DA, Keisari MS, et al (2013) Phylogenetic Findings Suggest Possible New Habitat and Routes of Infection of Human Eumyctoma. PLoS Negl Trop Dis 7: e2229. 23696914 10.1371/journal.pntd.0002229 PMC3656121

[20] Ahmed SA, van de Sande WWJ, Stevens DA, Fahal A, van Diepeningen A, et al (2014) Revision of agents of black-grain eumycetoma in the order *Pleosporales* Persoonia. 33: 141–154. 10.3767/003158514X684744 PMC4312930 25737597

[21] Möller EM, Bahnweg G, Sandermann H, Geiger HH (1992) A simple and efficient protocol for isolation of high molecular weight DNA from filamentous fungi, fruit bodies, and infected plant tissues. Nucleic Acids Res 20: 6115–6116. 1461751 10.1093/nar/20.22.6115 PMC334490

[22] Gerrits van den Ende AHG, De Hoog GS (1999) Variability and molecular diagnostics of the neurotropic species *Cladophialophora bantiana* . Stud Mycol 43: 152–162.

[23] Zhou X, Kong F, Sorrell TC, Wang H, Duan Y, et al (2008) Practical method for detection and identification of *Candida*, *Aspergillus*, and *Scedosporium* spp. by use of rollingcircle amplification. J Clin Microbiol 46: 2423–2437. 18495860 10.1128/JCM.00420-08 PMC2446935

[24] WHO (2013) The 17 neglected tropical diseases. Geneva: World Health Organization. Availabe: http://www.who.int/neglected_diseases/diseases/en/. Accessed 10 Nov 2014.

[25] Ahmed AO, Mukhtar MM, Kools-Sijmons M, Fahal AH, de Hoog S, et al (1999) Development of a species-specific PCR-restriction fragment length polymorphism analysis procedure for identification of *Madurella mycetomatis* . J Clin Microbiol 37: 3175–3158. 10488173 10.1128/jcm.37.10.3175-3178.1999 PMC85521

[26] de Hoog GS, van Diepeningen AD, Mahgoub el S, van de Sande WW (2012) New species of *Madurella*, causative agents of black-grain mycetoma. J Clin Microbiol 50: 988–994. 22205798 10.1128/JCM.05477-11 PMC3295137

[27] Hamzehei H, Yazdanparast SA, Davoudi MM, Khodavaisy S, Golehkheyli M, et al (2013) Use of rolling circle amplification to rapidly identify species of *Cladophialophora* potentially causing human infection. Mycopathologia 175: 431–438. 23471533 10.1007/s11046-013-9630-7

[28] Kong F, Tong Z, Chen X, Sorrell T, Wang B, et al (2008) Rapid identification and differentiation of *Trichophyton* species, based on sequence polymorphisms of the ribosomal internal transcribed spacer regions, by rolling-circle amplification. J Clin Microbiol 46: 1192–1199. 18234865 10.1128/JCM.02235-07 PMC2292936

[29] Ali MM, Li F, Zhang Z, Zhang K, Kang DK, et al (2014) Rolling circle amplification: a versatile tool for chemical biology, materials science and medicine. Chem Soc Rev 43: 3324–3341. 24643375 10.1039/c3cs60439j

[30] Nilsson M, Krejci K, Koch J, Kwiatkowski M, Gustavsson P, et al (1997) Padlock probes reveal single-nucleotide differences, parent of origin and in situ distribution of centromeric sequences in human chromosomes 13 and 21. Nat Genet 16: 252–255. 9207789 10.1038/ng0797-252

[31] Blanco L, Bernad A, Lázaro JM, Martín G, Garmendia C, et al (1989) Highly efficient DNA synthesis by the phage phi Ф 29 DNA polymerase. Symmetrical mode of DNA replication. J Biol Chem 264: 8935–8940. 2498321

[32] Banér J, Nilsson M, Mendel-Hartvig M, Landegren U (1998) Signal amplification of padlock probes by rolling circle replication. Nucleic Acids Res 26: 5073–5078. 9801302 10.1093/nar/26.22.5073 PMC147976

