# Supplementary figures and images for: Rapid Identification of Black Grain Eumycetoma Causative Agents Using Rolling Circle Amplification
Source: PLoS Negl Trop Dis. 2014 Dec 4;8(12):e3368. doi: 10.1371/journal.pntd.0003368 (PMC4256478; doi:10.1371/journal.pntd.0003368)

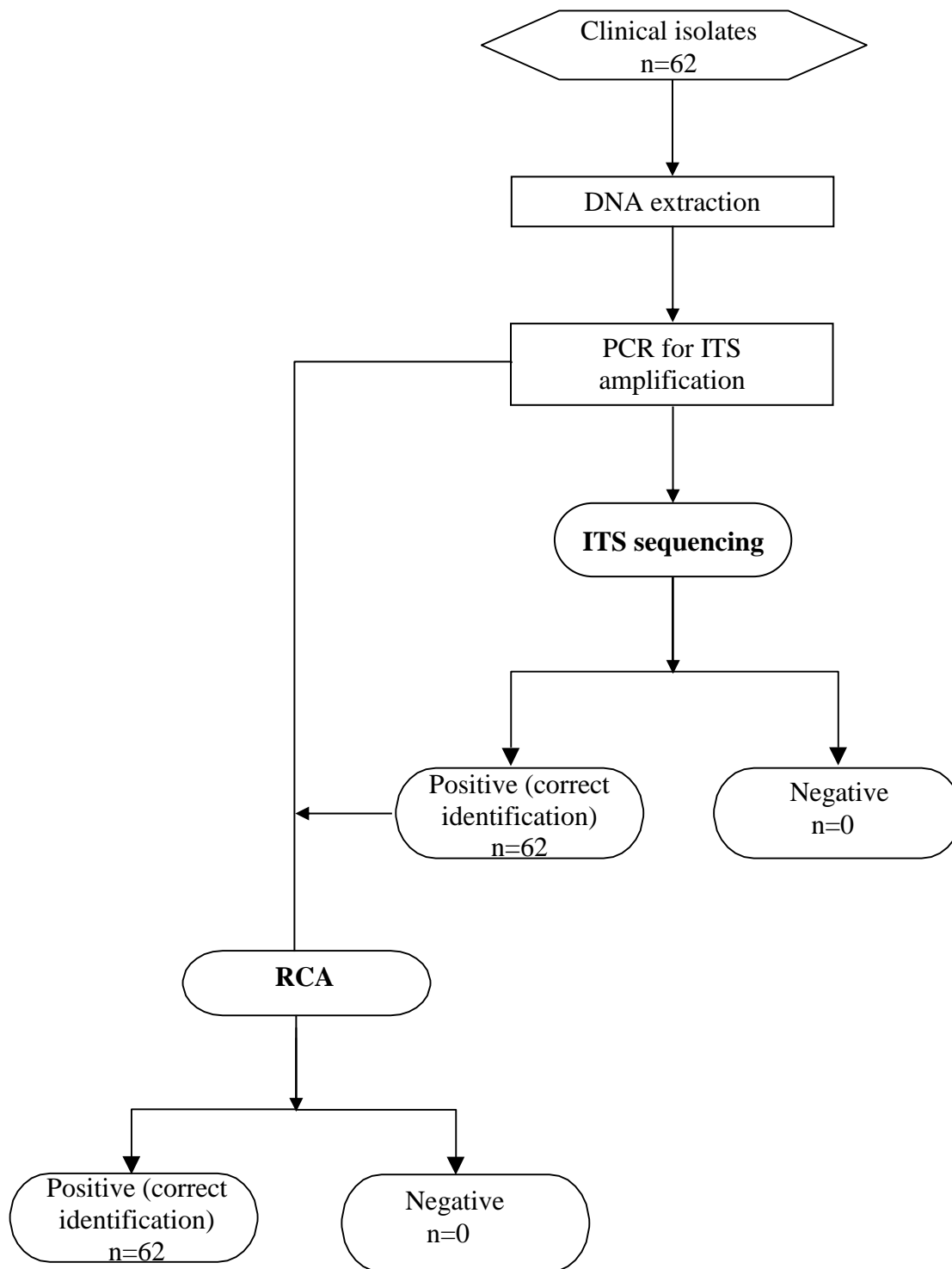

Supplement: S1 Figure — STARD flowchart for RCA. (PDF) [file pntd.0003368.s001.pdf]
